# Solvatochromism of Amphiphilic Au_25_(SR)_18_ Nanoclusters Based on Supramolecular Ligand–Thiolated Crown Ether

**DOI:** 10.1021/acs.jpclett.5c01543

**Published:** 2025-07-12

**Authors:** Patryk Obstarczyk, Subhradip Kundu, Thomas Bürgi, Joanna Olesiak-Bańska

**Affiliations:** † Institute of Advanced Materials, Wroclaw University of Science and Technology, Wybrzeże Stanisława Wyspiańskiego 27, Wrocław 50-344, Poland; ‡ Département de Chimie Physique, 27212 Université de Genève, 30 Quai Ernest Ansermet, CH-1211 Genève 4, Geneva, Switzerland

## Abstract

Atomically precise nanoclusters find multiple applications in, for example, bioimaging or as catalysts due to their remarkable molecule-like properties which are tightly bound to their strictly defined structures. However, their physicochemical characterization and broad utilization in the aforementioned areas are often challenging due to the limited solubility. Herein, we report the synthesis of Au_25_ nanoclusters (NCs) capped with 2-mercaptomethyl-12-crown-4 ether, imparting amphiphilic properties that confer solubility in both polar and nonpolar solvents. UV–vis spectroscopy confirmed the stability of the Au_25_ framework in polar protic and apolar aprotic solvents. Moreover, FTIR analysis suggests that our supramolecular ligand responds to solvent polarity and protic/aprotic conditions, adjusting its conformation on the cluster surface. Furthermore, accompanying variations in photoluminescence underscore their potential utility as environmentally responsive polarity-sensitive probes. These findings establish a versatile Au_25_ nanocluster platform for investigating structure–property relationships in diverse chemical environments without requiring additional chemical modifications to the cluster structure.

## Graphical abstract

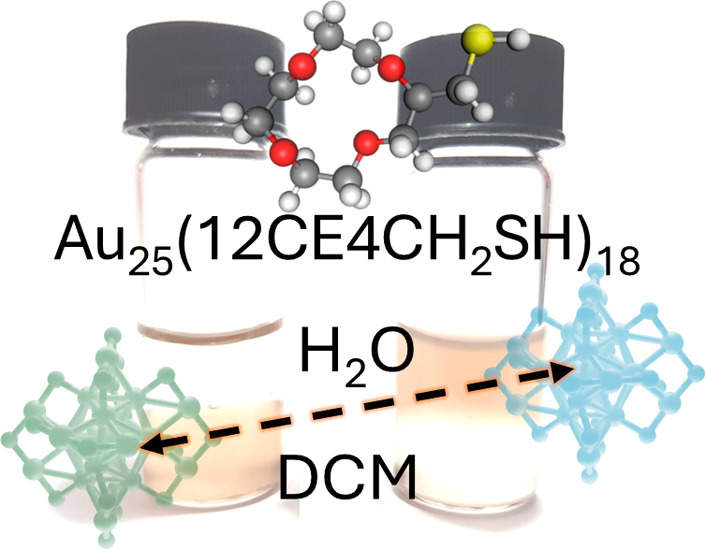

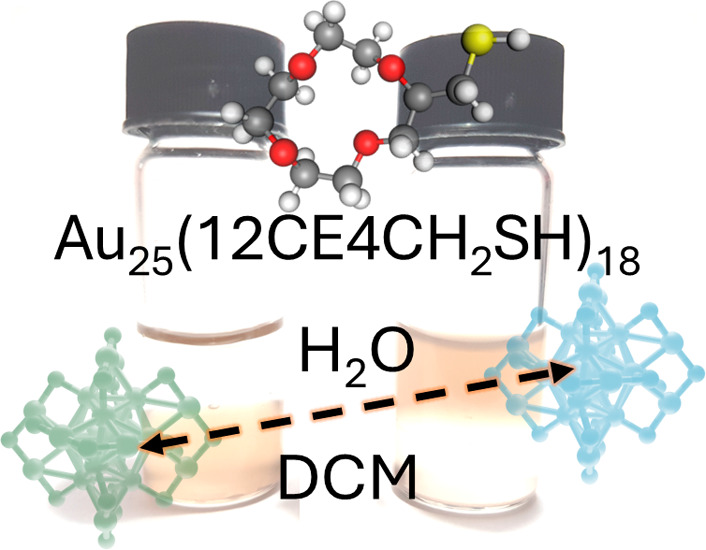

Atomically precise thiolate protected gold nanoclusters (NCs) are known as a group of nanostructures with sizes comparable to the Fermi wavelength of electrons in gold (i.e., below 2 nm) and unique molecular architectures.^1^ A variety of NC structures can be described within the Au_*n*_(SR)_*m*_ formula, where SR stands for thiolate ligand.^2^ NCs are hierarchically organized and composed of three structural components, i.e., (i) inorganic core, (ii) metal–ligand staple-like motifs, and (iii) protecting ligand shell.^3^ Due to their unique structure, NCs are well-known as molecule-like nanomaterials which bridge the gap between molecules and crystalline nanoparticles.^4^ In NCs, due to quantum confinement effects, discrete energy levels and a HOMO–LUMO gap emerge, which enable interesting photoluminescence properties^5,6^ and enhance their applicability in a variety of fields, including bioimaging.^7^ Moreover, the diversified photophysical properties of NCs can be further tuned due to the fact that even the smallest structural change in the cluster core or ligand shell composition can strongly modulate their electronic structure (every atom counts), thus resulting in the corresponding emission wavelength and quantum yield.^8^

The rapidly developing library of NCs allowed scientists to better understand structure–property correlations at the atomic level. However, the description of clusters’ chemical flexibility and their response to the environment, e.g., solvents, is not broadly explored.^9^ It is already established that the reaction medium plays a major role in influencing the final cluster size in a synthesis. Back in 2013, Liu et al.^10^ showed that Au­(I) intermediate aggregation state might be controlled by the solvent, which results in Au_25_ and Au_144_ clusters as synthesized upon reduction in tetrahydrofuran or methanol, respectively. Additionally, Li et al.^11^ indicated that Au_24_(S-TBBM)_20_ (S-TBBM, 4-tertbutylphenylmethancan) nanoclusters exhibit dual emission with a ratiometric response depending on solvent polarity. Analogously, Monti et al.^12^ showed that the solvent itself, namely, water, plays an active role in [Au_25_(GS)_18_]^−1^ (GS, glutathione) nanocluster optical activity, forming a chiral solvation shell around the cluster. In addition, clusters’ (i) core, (ii) staple-like motifs, and (iii) ligand shell structural distortions arising from weak interactions (including solvent effects) contribute to the modulation of their electronic properties.

Interestingly, most of the NCs available in the literature are limited in terms of their solubility. Cowan et al. performed theoretical studies and showed that a cluster’s symmetry (shape) and charge govern overall dipole moment and polarizability, which regulate interactions with solvents.^13^ However, none of the reported structures could be studied in a broad library of solvents, ranging from polar to apolar, without additional chemical modifications. The amphiphilicity of NCs was studied by Yao et al.^14^ and Shen et al.,^15^ but both methods were based on postsynthesis chemical functionalization of NCs with additional ligands, which, due to the presence of additional chemical and structural alterations, results in rather complex interactions between NCs and solvents. Therefore, simple systems, preferably based on inherently amphiphilic ligands, are highly desired candidates to study the chemical flexibility of Au_*n*_(SR)_*m*_ frameworks.

Within the spectrum of available molecular structures, cyclic oligomers of ethylene oxide, i.e., crown ethers (CEs), are classified as supramolecular compounds.^16^ Chemically, they are composed of 4 to 10 −CH_2_–CH_2_O– groups, which constitute a macrocycle (a ring-like structure). Moreover, due to their unique molecular architecture, CEs are characterized by a corresponding partition coefficient in octane water (*P* ^oct/water^) that varies depending on the number of ethylene oxide units in the macrocycle, ranging from 0.21 to 2.2, which makes them amphiphilic.^17^ Kunstmann-Olesen et al.^18^ already proved that CEs can work as a nanoscale shuttler in contact with membranes and are effective in the control over plasmonic nanoparticles’ hydrophobicity. The Brust group^19^ also studied plasmonic nanostructures capped with 18-crown-6 ether and presented temperature-dependent spontaneous agglomeration of nanoparticles due to the hydrophobic interactions. Our group has recently shown that crown ethers can be a functional ligand for ultrasmall nanoclusters, which provide the possibility of multimodal imaging of biological samples under fluorescence and transmission electron microscopy.^20^ However, previously reported structures were not atomically precise.

To address the issues presented above, in this work, we show the synthesis and characterization of fluorescent and amphiphilic Au_25_ NCs stabilized with 2-(mercaptomethyl)-12-crown-4 ether (12CE4CH_2_SH, for short). Our Au_25_(12CE4CH_2_SH)_18_ can be, without any additional modifications, well-solubilized in a variety of polar and apolar solvents, from water to toluene. We observed that for such an amphiphilic Au_25_ its photoluminescence maxima are located in the NIR region and remain unaltered in various solvents. However, its quantum yield is solvent-dependent and, in comparison to anionic Au_25_(C_6_H_5_CH_2_CH_2_SH)_18_ (Au_25_(PET)_18_ for short), is 2.08 times higher. We show that the photoluminescence lifetime of Au_25_(12CE4CH_2_SH)_18_ does not change linearly with solvent polarity index in protic solvents, whereas linear behavior was observed for aprotic solvents. In terms of the characteristic absorption bands of Au_25_(12CE4CH_2_SH)_18_, a small blue shift, i.e., ∼10 nm, can be observed for clusters dissolved in water, in comparison to dichloromethane. Our FITR studies confirmed that the conformation of the 12CE4CH_2_SH changes in response to the proticity of solvents used, as indicated by shifts and broadening in the C–O–C stretching region (∼1127 cm^–1^).

The 12CE4CH_2_SH-capped NCs were synthesized as described in the Supporting Information, transferred from water to dichloromethane, concentrated, and passed through a 1 m long column filled with styrene divinylbenzene beads. Due to the size exclusion, several fractions were separated and characterized based on their UV–vis–NIR spectra (Figure S1). Based on UV–vis–NIR spectral characteristics, fraction no. 5 was chosen for further studies and will be referred to as Au_25_(12CE4CH_2_SH)_18_ from now on. To support our claims regarding the atomically precise nature of our synthesis product, several techniques were utilized, as described below.

The structural properties of Au_25_(12CE4CH_2_SH)_18_ NCs were studied under TEM, as shown in Figure S2. The overall average diameter of the CE-capped NCs is equal to 1.8 ± 0.2 nm. To study the molecular composition of our CE-capped NCs and support our claims of their atomically precise nature, MALDI-TOF MS^21^ was utilized (see Figure S3). The MALTI-TOF MS peak at 8920.4 *m*/*z* is characteristic of Au_25_(12CE4CH_2_SH)_18_, as the molecular weight of 12-(mercaptomethyl)-12-crown-4 ether equals 222.3 AMU. The presence of the atomically precise structure, i.e., Au_25_(SR)_18_ framework, was thus confirmed.

The UV–vis–NIR spectrum of our as-separated product is presented in Figure 1, in comparison with broadly studied references (i.e., [Au_25_(PET)_18_]^−^[TOA]^+^ (anionic), and its oxidized form [Au_25_(PET)_18_]^0^ (neutral)).

**Figure 1:**
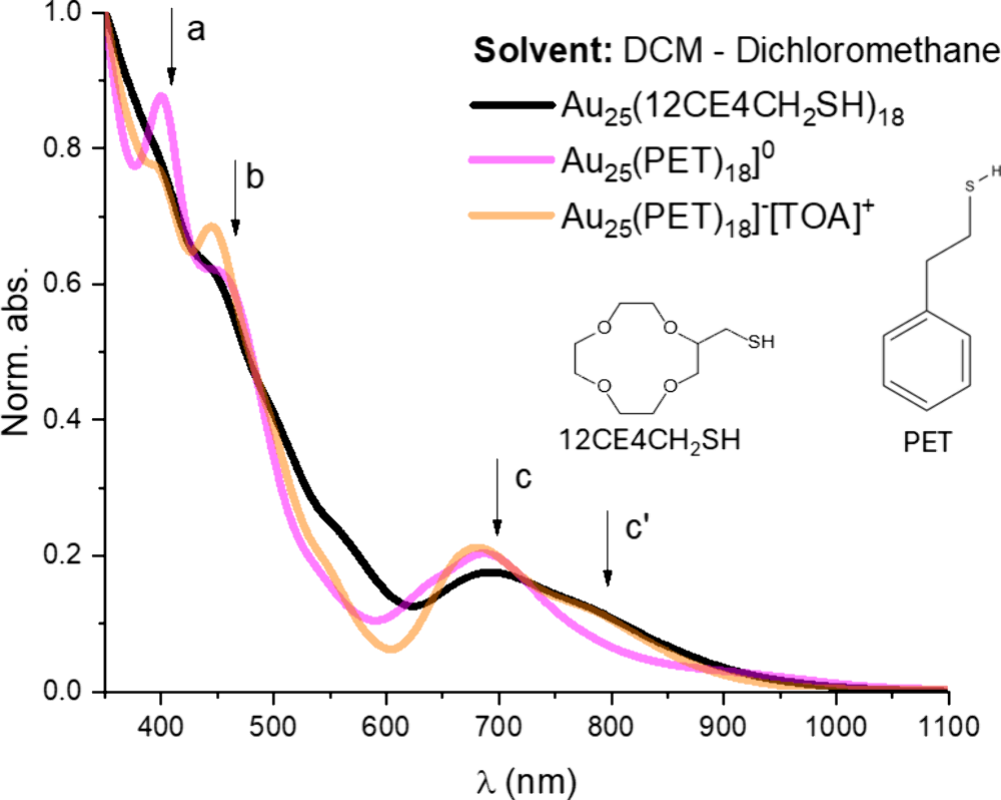
(a) Normalized optical absorbance spectra of Au_25_(12CE4CH_2_SH)_18_ NCs (black line) and its corresponding reference stabilized with 2-phenylethyl mercaptan (C_6_H_5_CH_2_CH_2_SH, PET), as measured in dichloromethane: pink line, [Au_25_(PET)_18_]^0^ - oxidized (neutral) form; orange line, [Au_25_(PET)_18_]^−^[TOA]^+^ - reduced (anionic) form with counterion. Characteristic electronic transitions of the Au_25_ framework are labeled as a (∼425 nm), b (∼450 nm), c (∼700 nm), and c′ (∼800 nm). Insets show the molecular structures of both ligands: 2-phenylethyl mercaptan (PET) and 2-(mercaptomethyl)-12-crown-4 ether (12CE4CH_2_SH).

For Au_25_(12CE4CH_2_SH)_18_, characteristic bands at ∼425 (a), ∼450 (b), ∼700 (c) and ∼800 (c′) nm (Figure 1, black line) were identified. These bands are in a good agreement with the calculations conducted for the Au_25_(SR)_18_ framework with a variety of ligands,^22^ as well as with the experimentally measured [Au_25_(PET)_18_]^0^ and [Au_25_(PET)_18_]^−^[TOA]^+^ NCs.^23^ Therefore, it supports that our product is Au_25_(12CE4CH_2_SH)_18_. Interestingly, in comparison to PET-capped NCs, a red shift of the c band was observed. The shift is equal to 5 and 11 nm, with respect to the neutral and anionic reference NCs, respectively (λ_12CE4CH_2_SH_ = 693 nm, λ_PET_ neutral = 688 nm, λ_PET_ anionic= 682 nm). It was already established that the HOMO–LUMO gap may change due to distortions of the Au_13_ icosahedral core of Au_25_(SR)_18_ framework or staple-like motifs derived from, for example, a ligand induced surface charge anisotropy.^24^ The oxidation state of Au_25_(12CE4CH_2_SH)_18_ clusters cannot be unambiguously determined based on UV–vis–NIR spectra alone; however, due to the presence of so-called “shoulder band” ^23,25^ (c′, ∼800 nm) the anionic character is expected.

12-Crown-4 ether is already identified as an amphiphilic molecule, and its partition coefficient is equal to 0.92, as determined for the mixture of 1-octanol and water.^17,26^ In our previous work^20^ we already showed that amphiphilic properties of crown ethers can be successfully transferred onto nonatomically precise nanostructures. Therefore, amphiphilicity is expected in Au_25_(12CE4CH_2_SH)_18_ NCs. As shown in Figure S4, Au_25_(12CE4CH_2_SH)_18_ can be readily dissolved in water. However, when a nonmiscible (in reference to water) phase is added, a spontaneous phase-transfer of NCs from aqueous to organic solvent is observed within 1 h. Such phenomenon corresponds to the 12-crown-4 ether physicochemical properties, as its partition coefficient suggests that it has slightly higher affinity to organic solvents.^17^ In order to test the hypothesis of amphiphilicity of Au_25_(12CE4CH_2_SH)_18_, several solvents were chosen to study the solubility of our NCs (Figure 2a).

**Figure 2:**
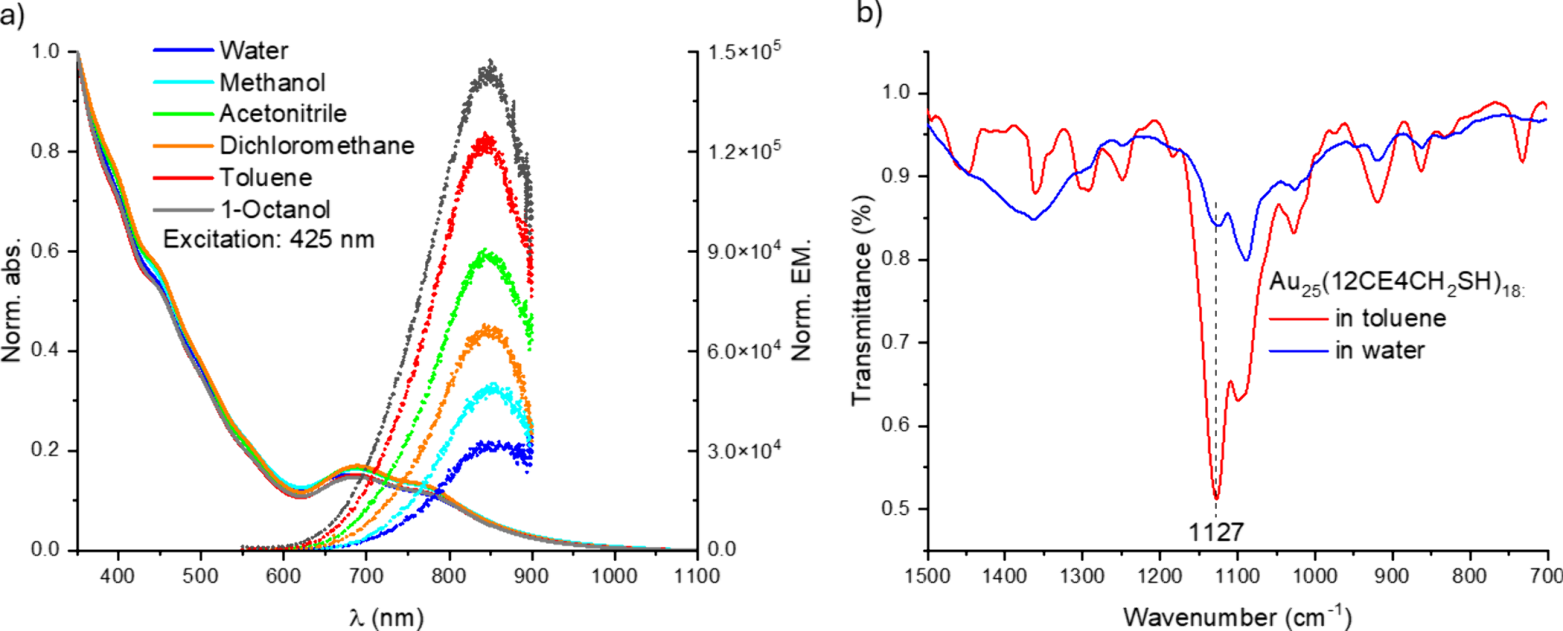
(a) Normalized optical absorbance (solid lines) and emission spectra (dotted lines) of Au_25_(12CE4CH_2_SH)_18_ in a variety of solvents: from polar protic (e.g., H_2_O) to apolar aprotic (e.g., toluene). Emission spectra normalization was performed by dividing the entire emission spectra by the clusters absorbance at excitation wavelength, namely, at 425 nm, as registered in respective solvents. (b) FTIR spectra of Au_25_(12CE4CH_2_SH)_18_ in toluene (red line) and water (blue line). The characteristic C–O–C stretching wavenumber of CE is marked with a black dashed line.

In detail, Au_25_(12CE4CH_2_SH)_18_ NCs were dissolved in dichloromethane; divided into 6 bottles; dried under reduced pressure; and subsequently redissolved in water, methanol, acetonitrile, dichloromethane, toluene, and 1-octanol. The corresponding absorbance spectra of Au_25_(12CE4CH_2_SH)_18_ nanoclusters were then registered in the aforementioned solvents and are presented in Figure 2a, together with their respective luminescence spectra. The corresponding experiment was also performed for [Au_25_(PET)_18_]^0^ nanoclusters (Figure S5); however, as the PET-capped clusters are not soluble in polar solvents, it was not possible to obtain the corresponding spectra in, for example, water and methanol. As Au_25_(12CE4)_18_ could be readily dissolved in a range of solvents, our atomically precise nanostructures based on crown ethers are indeed amphiphilic. Moreover, the structural features of Au_25_(SR)_18_ are maintained in every solvent, as characteristic peaks (a, b, c, c′) are consistently present. However, a small blue shift of the c′ band, i.e., ∼10 nm, can be observed for Au_25_(12CE4CH_2_SH)_18_ dissolved in water, in comparison to dichloromethane. On the other hand, for Au_25_(12CE4CH_2_SH)_18_, no shifts were observed between aprotic and polar (acetonitrile) and aprotic apolar (toluene). These two aprotic solvents are also suitable for [Au_25_(PET)_18_]^0^, where, similar to the previous case, no spectral shifts were recorded. Shifts observed for Au_25_(12CE4CH_2_SH)_18_ between water and dichloromethane might be therefore explained by structural distortions induced by crown ethers subjected to solvents of protic nature (hydrogen bond network formation),^27,28^ stabilization of electronic states by the polar solvents, as well as charge tuning in halogenated solvents.^29^

Interestingly, as shown in Figure 2a, Au_25_(12CE4CH_2_SH)_18_ cluster photoluminescence (PL) maxima positions remain unaltered upon dissolution in various solvents, but as the PL falls in the NIR region, where the detectors have limited sensitivity, the subtle shifts in PL maxima may not be detected. However, the collected PL intensity is sufficient to determine PL quantum yields (QY) for different solvents. Au_25_(12CE4CH_2_SH)_18_ PL QY range from 0.2 to 1.1%, as presented in Table 1. These values are in a good agreement with data presented in the literature for the Au_25_(SR)_18_ framework, where no additional chemical modifications are present.^6,30^ However, in comparison to Au_25_(PET)_18_ in toluene (QY = 0.48% for anionic clusters),^31^ the QY of CE-capped NCs in the corresponding solvent is 2.08 times higher. The observed PL intensity variations as registered between distinctive solvents may be also attributed to quenching effects arising from solvent penetration propensity through the ligand shell, as was already demonstrated in the optical properties of zwitterion functionalized gold nanoclusters by performing quantum mechanics/molecular mechanics (QM/MM) simulations.^32^ For deeper insight into the PL properties of Au_25_(12CE4CH_2_SH)_18_ clusters, we determined PL decay times in distinctive solvents, as presented in Table 1.

**Table 1:** Quantum Yield (QY) and Average Photoluminescence Lifetime of Au_25_(12CE4CH_2_SH)_18_ Nanoclusters in Protic and Aprotic Solvents Ranked According to Their Respective Polarity Index (PI)

PI	Solvent	QY (%)	⟨τ_verage_⟩ (ns)
**Protic**			
9.0	Water	0.2	13971 ± 604
5.8	Methanol	0.34	20839 ± 903
4.3	1-Octanol	1.1	2047 ± 50
**Aprotic**			
5.1	Acetonitrile	0.71	1861 ± 143
3.1	Dichloromethane	0.57	1660 ± 17
2.1	Toluene	1	1479 ± 31

In both protic and aprotic solvents, polarity modulates the average PL lifetimes of our clusters (Table 1, Table S1, Figure S6), which range from 20839 to 2047 and from 1861 to 1479 ns, in protic and aprotic solvents, respectively. Overall, for more polar solvents the corresponding PL lifetimes are longer. However, in terms of protic solvents, the lifetimes do not change linearly with solvent polarity index. For Au_25_(12CE4CH_2_SH)_18_ NCs in methanol, in comparison to water and octanol, the PL lifetime is 1.5 times longer and 10 times shorter, respectively. Such a phenomenon might relate to the differences in possible conformational landscapes of our supramolecular compounds in water compared to methanol.^27,33^ Therefore, not only the polarity of a solvent but also its ability to form hydrogen bonds is of great importance because it determines the NC’s nearest environment and influences the optical properties.

We hypothesize that the dependence of the emission and absorption spectra on the solvent is connected to conformational changes of crown ethers in different solvents. Such conformational changes were previously observed for crown ethers studied via FTIR studies.^33,34^ Thus, we measured FITR spectra of Au_25_(12CE4CH_2_SH)_18_ NCs in two representative solvents, i.e., toluene and water, as they represent apolar aprotic and polar protic solvents, respectively. As shown in Figure 2b, FTIR of Au_25_(12CE4CH_2_SH)_18_ in toluene and water are different, which reflects the different conformation of the ligand due to different interactions with the solvent. In toluene (red line), the sharp and intense C–O–C stretching band at 1127 cm^–1^ indicates minimal solvent interactions, as a well-preserved and rigid crown ring structure is expected to produce such a signal.^35^ In contrast, the FTIR spectrum of water (blue line) shows the C–O–C band, which is shifted to lower wavenumbers, which may be explained by hydrogen bonding between the ether oxygen atoms and water molecules.^27,33^ Such a phenomenon is responsible for the elongation and weakening of the C–O bond, implying complexation and increased conformational flexibility of the crown ether in the sample. The data highlight that solvent polarity and hydrogen bonding capacity significantly influence the structure of CE ligands bound to the Au_25_ NCs framework.

In this work we presented the synthesis and characterization of an amphiphilic Au_25_(12CE4CH_2_SH)_18_ nanocluster framework. The supramolecular ligand utilized in our studies, i.e., 2-mercaptomethyl-12-crown-4 ether, allows our nanoclusters to be readily solubilized in both polar and apolar solvents. Our cluster emission maxima is located in the NIR region and remains unaltered in every solvent used. However, the QY of our clusters is solvent-dependent and increases with a polarity index, ranging from 0.2 to 1.1%. The photoluminescence lifetime of Au_25_(12CE4CH_2_SH)_18_ ranges from 20839 to 2047 and from 1861 to 1479 ns, in protic and aprotic solvents, respectively. Interestingly, for aprotic solvents it is gradually increasing with a polarity index, which is not the case for protic solvents. Such unique changes of Au_25_(12CE4CH_2_SH)_18_ properties observed for protic and aprotic environments might be attributed to the CE ligand conformational landscape, which based on our FITR studies, is altered in response to solvents used. The higher affinity of our nanocluster system to organic solvents, in comparison to water, and their changes in photoluminescence lifetimes also imply their potential applicability in bioimaging as polarimetric probes, especially in terms of fluorescence lifetime-based microscopy. Overall, the design of atomically precise structures stabilized with an amphiphilic ligand being able to be solvated in a variety of solvents without additional chemical modifications is an important step for in-depth understanding of diverse effects at the nanoscale (e.g., solvatochromism) previously hampered by limited solubility of atomically precise nanostructures.

## Supplementary Material



## Notes

Experimental details: (1) list of chemicals and consumables used throughout the study, (2) Au_25_(12CE4CH_2_SH)_18_ synthesis and purification protocol, (3) [Au_25_(PET)_18_]^−^[TOA]^+^ synthesis and oxidation to [Au_25_(PET)_18_]^0^ protocol, (4) TEM imaging, MALDI-TOF-MS, FTIR, absorption, QY and fluorescence lifetime measurement protocols; optical absorbance spectra of 12-crown-4-CH_2_SH ether-capped nanoclusters fractions obtained via size exclusion chromatography; TEM image of Au_25_(12CE4CH_2_SH)_18_ nanoclusters; MALDI-TOF MS spectra of Au_25_(12CE4CH_2_SH)_18_; digital photographs of phase transfer of Au_25_(12CE4CH_2_SH)_18_ nanoclusters from water to dichloromethane; optical absorbance spectra of [Au_25_(PET)_18_]^0^ in variety of solvents; exemplary fluorescence lifetime measurements and fitting curves and corresponding fluorescence lifetimes percentage contributions (PDF)
