# Supplementary material for: Solvatochromism of Amphiphilic Au25(SR)18 Nanoclusters Based on Supramolecular Ligand–Thiolated Crown Ether
Source: J Phys Chem Lett. 2025 Jul 12;16(29):7331–6. doi: 10.1021/acs.jpclett.5c01543 (PMC12302204; doi:10.1021/acs.jpclett.5c01543)
Supplement: Supplementary file 1 [file jz5c01543_si_001.pdf]

Solvatochromism of amphiphilic of $Au_{25}(SR)_{18}$ nanoclusters based on supramolecular ligand – thiolated crown ether

Patryk Obstarczyk[1], Subhradip Kundu[2], Thomas Bürgi[2], Joanna Olesiak-Bańska[1*]

1 - Institute of Advanced Materials, Wroclaw University of Science and Technology, Wybrzeże Stanisława Wyspiańskiego 27, Wrocław 50-344, Poland

2 - Département de Chimie Physique, Université de Genève, 30 Quai Ernest Ansermet, CH-1211 Genève 4, Geneva,Switzerland

[*] - corresponding author – joanna.olesiak-banska@pwr.edu.pl

Experimental section

Chemicals and consumables

All chemical and materials utilized in this work were used as purchased. Tetrachloroauric (III) acid (99,9%, $HAuCl_4$ x 3 H_2O), sodium borohydride (≥98.0%, $NaBH_4$), sodium hydroxide (≥98%, NaOH), tetraoctylammonium bromide (98%, TOABr), 2-Phenylethyl mercaptan (98%, PET) as well as all organic solvents (HPLC grade) – *i.e.* methanol, acetonitrile, dichloromethane, toluene, 1-octane and tetrahydrofuran - were sourced by Merck. High-purity water (Mili-Q) was used throughout all the experiments. The stationary phase (SX1 BioBeds - styrene divinylbenzene - 1% cross-linkage, 40–80 µm bead size, 600–14000 MW exclusion range) was bought from Bio-Rads. The supramolecular ligand, namely 2-(mercaptomethyl)-12-crown-4 ether (> 95%), was supplied by ProchimiaSurfaces.

Au_{25}(12CE4CH$_2$SH)$_{18}$ nanoclusters synthesis

Au_{25}(12CE4CH$_2$SH)$_{18}$ nanoclusters were prepared by the dropwise addition of 1.5 mL of $NaBH_4$ (20 mM in aqueous solution of 0.5 M NaOH) to a solution composed of (1) 40 mL of water, (2) 2.5 mL of $HAuCl_4 \cdot 3H_2O$ (20 mM, in water), (3) 0.222 mL of 2-(mercaptomethyl)-12-crown-4 ether (0.45 M, in methanol), and (4) 2.5 mL of NaOH (0.1 M, in water) under slow stirring (250 rpm). During the reduction, the mixture changed color from pale yellow to orange-brown and was left undisturbed for 48 hours. In the next step, 40 mL of dichloromethane was added to the reaction flask. After some time, the aqueous phase became colorless and the organic phase turned orange-brown. The basic aqueous phase was discarded, while the organic phase was concentrated using a rotary evaporator. The concentrated sample was subsequently purified using a SEC column (stationary phase: SX1 BioBeads, styrene-divinylbenzene), and the fraction containing Au_{25}(12CE4CH$_2$SH)$_{18}$ nanoclusters was separated for future studies.

$[Au_{25}(PET)_{18}]^{-1}[TOA]^{+}$ (anionic form) synthesis and oxidation to $[Au_{25}(PET)_{18}]^{0}$ (oxidized, neutral form)

Nanoclusters capped by PET were synthesized based on previously described protocols[1]. In short, $[Au_{25}(PET)_{18}]^{-1}$ clusters were synthesized by reduction of $HAuCl_4$ x 3 H_2O (1g) and TOABr (1.641 g) complex by sodium borohydride $NaBH_4$ (0.917g, 50 mL aqueous),

in THF. The product of the reaction, after 48 h of aging, was dried under a rotary evaporator and washed with methanol (~ 2 L) to remove unreacted thiols. The resulting powder (i.e. $[Au_{25}(PET)_{18}]^{-1}[TOA]^{+}$) was dissolved in toluene and passed through a SEC column (stationary phase: SX1 BioBeads, styrene divinylbenzene). After that, chosen fractions containing pure $[Au_{25}(PET)_{18}]^{-1}[TOA]^{+}$ (based on UV-Vis spectra) were collected and split in two equal parts by volumes. Second portion of $[Au_{25}(PET)_{18}]^{-1}[TOA]^{+}$ was then oxidized over a silica column with dichloromethane as a stationary phase. Neutral $[Au_{25}(PET)_{18}]^{0}$ was collected and subsequently purified over a second SEC column (stationary phase: SX1 BioBeads, styrene divinylbenzene).

TEM imaging

An aqueous solution of NCs was drop-casted onto a carbon-coated copper grid and dried. A TALOS F200i (ThermoFisher Scientific) operating at 200 kV electron source was used for imaging.

MALDI-TOF MS measurements

Spectra were recorded on a Bruker Autoflex Speed Mass Spectrometer in linear positive (LP) mode with a nitrogen laser. First stock solution of trans-2-[3-(4-tert-butylphenyl)-2-methyl-2-propenylidene] malononitrile (DCTB) matrix was prepared in DCM solvent with the concentration of 35 mg/ml. Matrix stock solution (5 µL) and the sample solution (5 µL of concentration 1 mg/ml) were mixed together at room temperature and 2 µL of the mixture was spotted on the MALDI plate and air-dried prior to recording the data.

Absorption, QY and fluorescence lifetimes

The UV−Vis-NIR spectra of nanoclusters were measured in a QS high-precision cell (10 mm, supplied by Hellma Analytics) using a JASCO V-670 spectrophotometer.

Fluorescence quantum yields were determined using a comparative method[2], with FS5 Spectrofluorometer (Edinburgh Instruments) equipped with a Xenon lamp and a JASCO V-670 spectrophotometer. Styryl 9M dye in chloroform (QY = 27.2 ± 2.0%) served as the standard – reference compound. Both, the standard and nanoclusters were excited at 425 nm. The corresponding QYs were calculated using the following equation (1, 2):

$$QY_S = QY_S \frac{Af_R}{AF_S} \frac{Int_S}{Int_R} \frac{n_S^2}{n_R^2} \tag{1}$$

$$Af = 1 - 10^{-A_\lambda} \tag{2}$$

where QY, Af, Int, A_λ and n denote the QY, absorption factor at excitation wavelength, integrated luminescence intensity, absorbance at excitation wavelength, and refractive index of the solvents, respectively. Subscript R stands for the reference compound, while S stands for sample.

Time-correlated single photon counting (TCSPC) was employed to collect fluorescence lifetime spectra using FS5 Spectrofluorometer (Edinburgh Instruments) with LED EPLED-450 (Edinburgh Instruments) excitation source. Decays were measured with 20 μs time range on 8192 channels (time/ch.: 2.441 ns) while emission wavelength was set at 820 nm, and excitation was fixed at 450 nm (with a 20 μs pulse period). Instrument response functions (IRF) was determined from the colloidal silica. To fit the data multiexponential model decay from Fluoracle software (Edinburgh Instruments) was applied (3):

$$R(t) = B_1 exp\left(-\frac{t}{\tau_1}\right) + B_2 exp\left(-\frac{t}{\tau_2}\right) + (\ldots) \tag{3}$$

Average lifetime of the entire fluorescence decay process was then calculated according to the formula (4):

$$< \tau_{average} >= \frac{B_1\tau_1^2 + B_2\tau_2^2 + (\ldots)}{B_1\tau_1 + B_2\tau_2 + (\ldots)} \tag{4}$$

where B_1, B_2 are the contribution coefficients of the particular lifetime components.

FTIR measurements

FTIR spectra were recorded on a FT-IR Bruker Optic GmbH Vertex 70v Raman spectrometer in a 700 – 1500 cm^{-1} wavenumber range, where highly concentrated nanoclusters samples (~ 10 mg/mL) were used. Measurement time range was adjusted to collect the data from solvated samples, which was monitored by presence or absence of characteristic signals assigned to water and toluene, respectively.

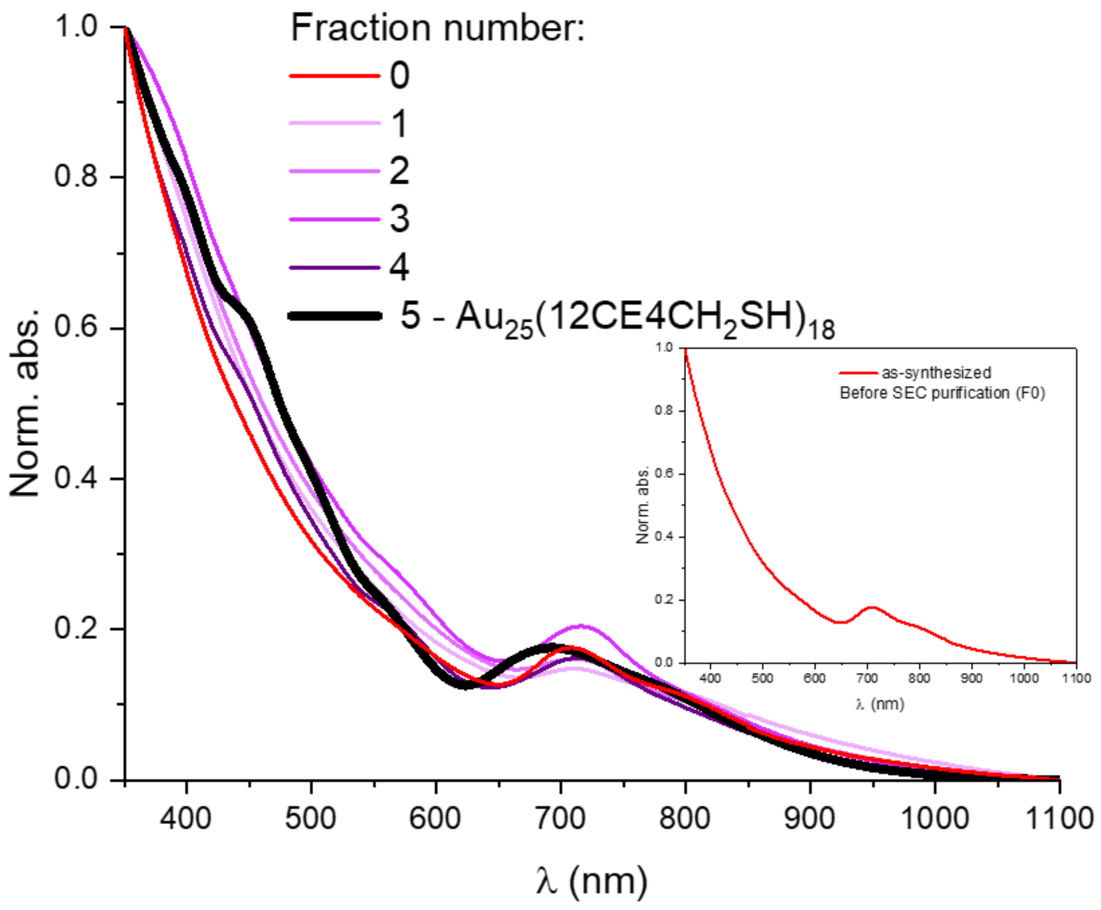

Fig. S1. Optical absorbance spectra of dichloromethane soluble fractions (1- 5) obtained *via* size exclusion chromatography on porous styrene divinylbenzene bead from as synthesized 12-crown-4-CH2CSH ether-capped nanoclusters mixture. Inset shows optical absorbance spectra of as-synthesized material, before SEC purification – fraction 0. Main product – $Au_{25}(12CE4CH_2SH)_{18}$ –, *i.e.* fraction 5, marked with bolded line.

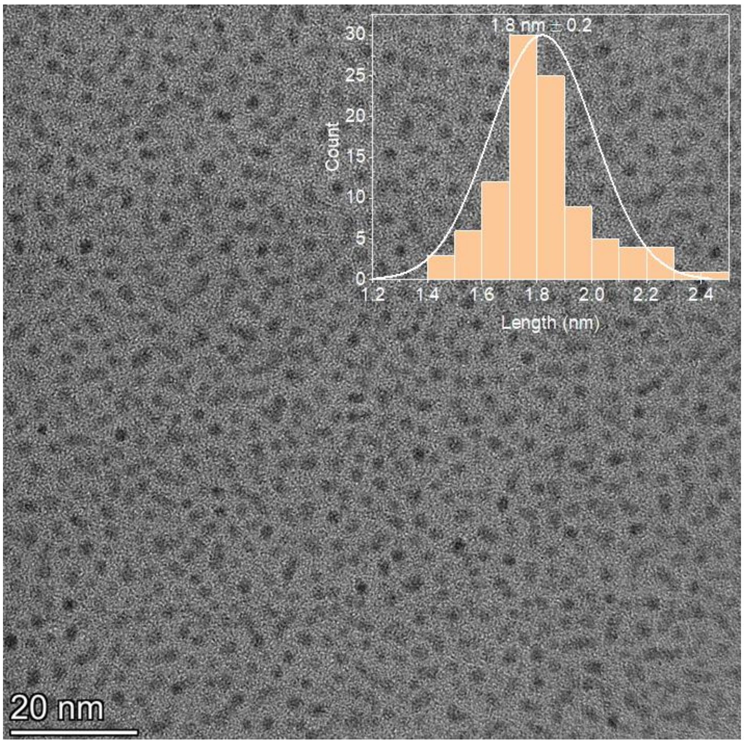

Fig. S2. Transmission electron microscope (TEM) image of dried $Au_{25}(12CE4CH_2SH)_{18}$ nanoclusters (NCs) drop-cast on carbon film-coated copper grids. The inset shows the statistical size distribution of the NCs based on the presented image, with a mean size of 1.8 ± 0.2 nm.

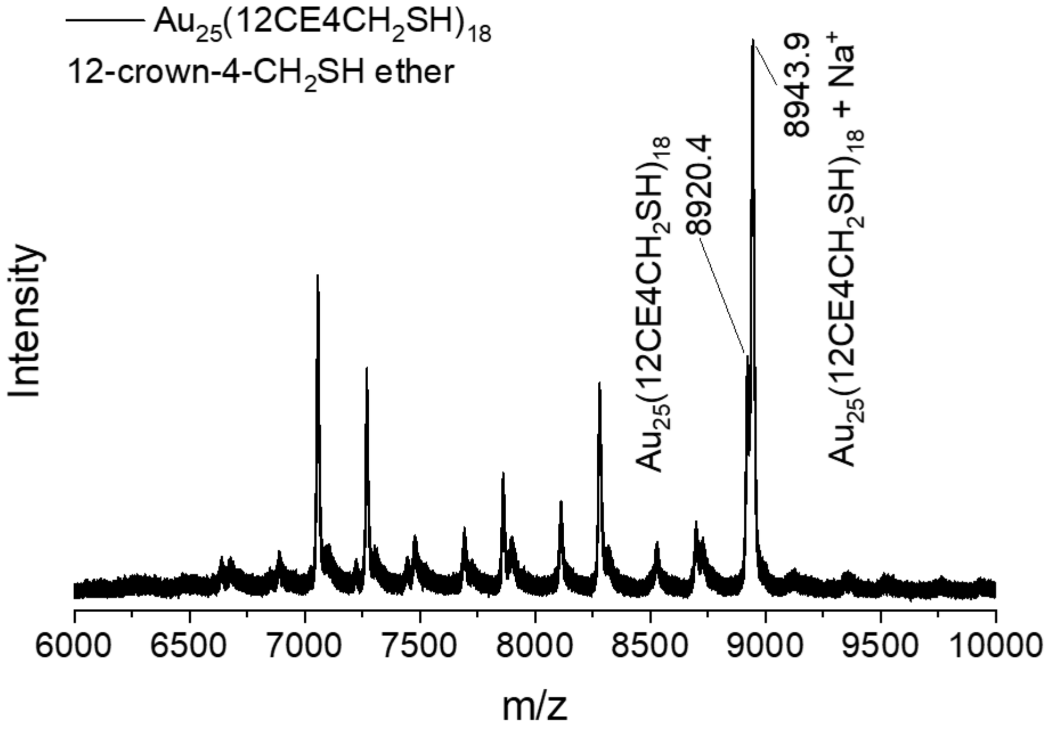

Fig. S3. MALDI-TOF MS spectra of $Au_{25}(12CE4CH_2SH)_{18}$ and $Au_{25}(12CE4CH_2SH)_{18} + Na^{+}$ with corresponding peaks at 8920.4 and 8943.9 m/z.

As shown in Fig. S3, two peaks, *i.e.* 8943.9 and 8920.4 m/z might be assigned to $Au_{25}(12CE4CH_2CSH)_{18}$. The former peak may come from $Au_{25}(12CE4CH_2CSH)_{18} + Na^+$, as sodium borohydride was utilized as a reducing agent in the synthesis protocol and crown ethers themselves can form complexes with alkaline metals ions[3]. Additional peaks from MALDI-TOF MS spectra cannot be unambiguously determined, as clusters can decompose and form fragments during mass spectrometric analysis[4]. However, obtained mass could not to be assigned to $Au_{25}(SR)_{18}$ L_xM_y fragments. We would like to emphasize that bulky ligand - crown ether - may also fragment due to the exposure to the laser source, which makes the identification of registered peaks challenging

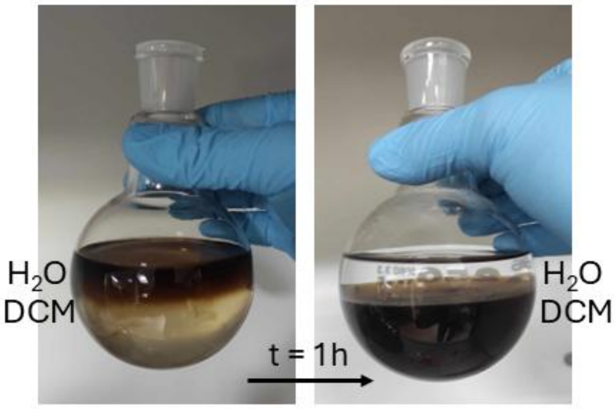

Fig. S4. Spontaneous and complete phase transfer of as-synthesized $Au_{25}(12CE4CH_2SH)_{18}$ nanoclusters from water to dichloromethane - 1:1 volume ratio (100 mL per solvent), 1h, no stirring, 25˚C. Complete phase transfer was confirmed by UV-Vis spectroscopy.

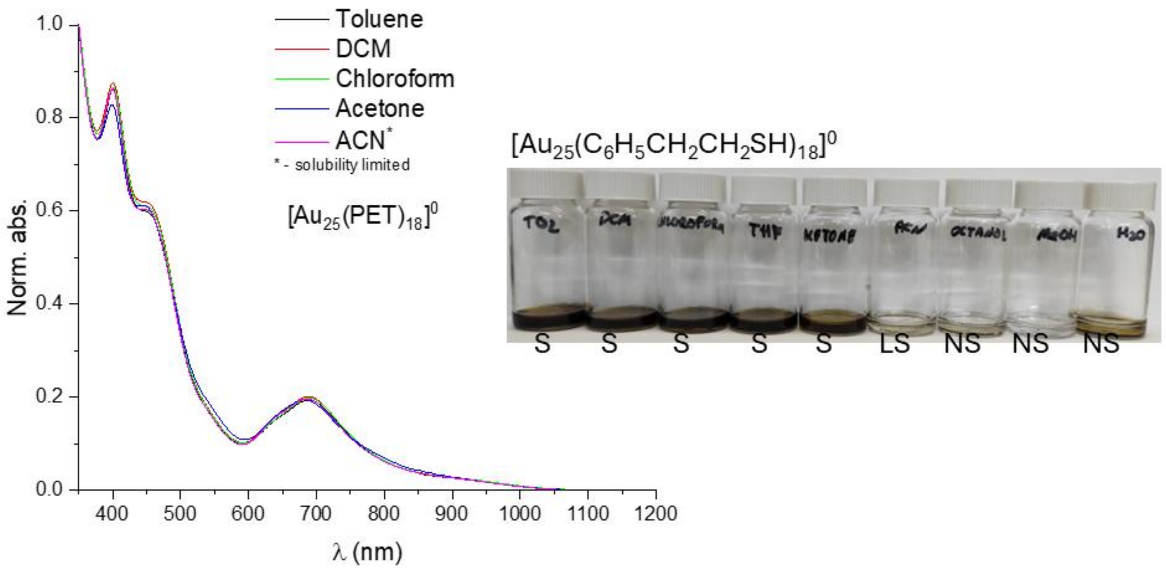

Fig. S5. Normalized optical absorbance spectra of $[Au_{25}(PET)_{18}]^0$ – oxidized (neutral) form – in variety of solvents. Inset show photography of solutions or unusable powders of $[Au_{25}(PET)_{18}]^0$ NCs in toluene, dichloromethane, chloroform, tetrahydrofuran, acetone, acetonitrile, methanol, water, from left to right, respectively. Descriptions refers to NCs solubility, as follow: S – soluble, LS – soluble with limitations, NS – not soluble.

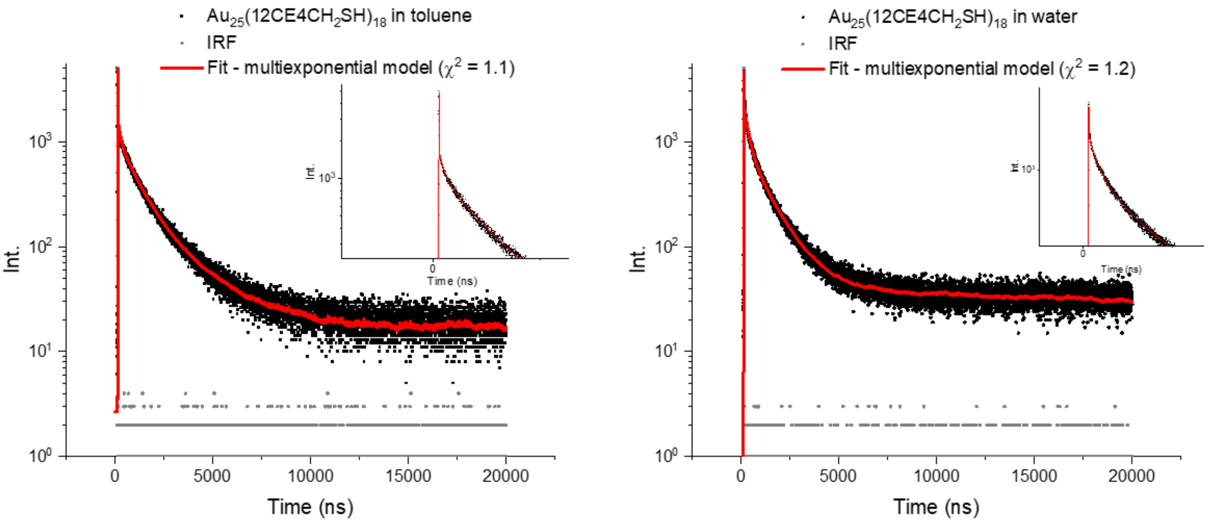

Figure. S6. Exemplary fluorescence lifetimes measurements and fitting curves as registered for $Au_{25}(12CE4CH_2SH)_{18}$ in water (right) and toluene (left).

Table S1. Fluorescence lifetimes percentage contributions - $Au_{25}(12CE4CH_2SH)_{18}$.

**Aprotic solvents**	**T_1 (ns)**	**%**	**τ_2 (ns)**	**%**	**τ_3 (ns)**	**%**	**τ_4 (ns)**	**%**	**<τ_{avr}> (ns)**
Acetonitrile	2.22 ± 2.23	88.09	307.25 ± 10.03	4.25	1323.78 ± 25.49	6.39	3444.57 ± 93.30	1.27	1861.33 ± 143.51
Dichloromethane	1.89 ± 0.07	87.66	119.08 ± 6.61	3.41	793.561 ± 16.38	5.43	2249.35 ± 19.20	3.50	1660.30 ± 17.67
Toluene	1.93 ± 0.06	90.95	140.01 ± 6.47	3.24	890.63 ± 19.09	4.37	2393.63 ± 41.29	1.44	1479.03 ± 31.98
**Protic solvents**	**T_1 (ns)**	**%**	**τ_2 (ns)**	**%**	**τ_3 (ns)**	**%**	**τ_4 (ns)**	**%**	**<τ_{avr}> (ns)**
Water	7.14 ± 0.18	70.89	237.01 ± 3.29	17.53	1137.95 ± 6.97	11.14	30830.99 ± 879.02	0.44	13971.66 ± 604.82
Methanol	2.14 ± 0.08	87.59	367.23 ± 5.92	5.92	1618.80 ± 11.31	6.07	36344.56 ± 1125.10	0.42	20839.68 ± 903.90
Octanol	2.69 ± 0.53	81.20	324.67 ± 7.60	7.32	1471.89 ± 17.40	10.19	4377.37 ± 102.11	1.29	2047.59 ± 50.36

References

(1) Parker, J. F.; Weaver, J. E. F.; McCallum, F.; Fields-Zinna, C. A.; Murray, R. W. Synthesis of Monodisperse [Oct4N+][Au25(SR)18−] Nanoparticles, with Some Mechanistic Observations. *Langmuir* **2010**, *26* (16), 13650-13654. DOI: 10.1021/la1020466. Zhu, M.;

Eckenhoff, W. T.; Pintauer, T.; Jin, R. Conversion of Anionic [Au25(SCH2CH2Ph)18]− Cluster to Charge Neutral Cluster via Air Oxidation. *The Journal of Physical Chemistry C* **2008**, *112* (37), 14221-14224. DOI: 10.1021/jp805786p.
(2) Rurack, K.; Spieles, M. Fluorescence Quantum Yields of a Series of Red and Near-Infrared Dyes Emitting at 600−1000 nm. *Analytical Chemistry* **2011**, *83* (4), 1232-1242. DOI: 10.1021/ac101329h.
(3) Shalit, Y.; Tuvi-Arad, I. Symmetry–Binding Correlations of Crown Ether Complexes with Li+ and Na+. *ACS Omega* **2021**, *6* (29), 19233-19237. DOI: 10.1021/acsomega.1c02684. Brzezinski, B.; Schroeder, G.; Rabold, A.; Zundel, G. H+, Li+, and Na+ Polarizabilities in 1:1 Crown Ether Cation Complexes. A FTIR Study. *The Journal of Physical Chemistry* **1995**, *99* (21), 8519-8523. DOI: 10.1021/j100021a012.
(4) Dass, A.; Stevenson, A.; Dubay, G. R.; Tracy, J. B.; Murray, R. W. Nanoparticle MALDI-TOF Mass Spectrometry without Fragmentation: Au25(SCH2CH2Ph)18 and Mixed Monolayer Au25(SCH2CH2Ph)18−x(L)x. *Journal of the American Chemical Society* **2008**, *130* (18), 5940-5946. DOI: 10.1021/ja710323t. Knoppe, S.; Bürgi, T. The fate of Au25(SR)18 clusters upon ligand exchange with binaphthyl-dithiol: interstaple binding vs. decomposition. *Physical Chemistry Chemical Physics* **2013**, *15* (38), 15816-15820, 10.1039/C3CP52634H. DOI: 10.1039/C3CP52634H.
